# Detecting the limits of the biological effects of far-infrared radiation on epithelial cells

**DOI:** 10.1038/s41598-019-48187-0

**Published:** 2019-08-12

**Authors:** Yung-Ho Hsu, Yu-Wei Chen, Chung-Yi Cheng, San-Liang Lee, Tzu-Hsuan Chiu, Cheng-Hsien Chen

**Affiliations:** 1 Department of Internal Medicine, School of Medicine, College of Medicine, Taipei Medical University, Taipei, Taiwan; 2 Division of Nephrology, Department of Internal Medicine, Shuang Ho Hospital, Taipei Medical University, New Taipei City, Taiwan; 3 Division of Nephrology, Department of Internal Medicine, Wan Fang Hospital, Taipei Medical University, Taipei, Taiwan; 4 Department of Electronic and Computer Engineering, National Taiwan University of Science and Technology, Taipei, Taiwan

**Keywords:** Assay systems, Cell migration

## Abstract

Far-infrared radiation (FIR) exerts numerous beneficial effects on health and cell physiology. Recent studies revealed that the biological effects of FIR are independent of thermal effects. There is no proper method for measuring the parameters of the non-thermal biological effects of FIR, which limits its biomedical application. In this study, we established a cell detection platform using epithelial cell migration to measure the limits of the biological effects of FIR. FIR promoted the migration of rat renal tubular epithelial cells as revealed by our standardized detection method. We defined the ratio of the FIR-promoted migration area to the migration area of the control group as the FIR biological index (FBI). An increase of the FBI was highly associated with FIR-promoted mitochondrial function. Through FBI detection, we revealed the limits of the biological effects of FIR, including effective irradiation time, wavelengths, and temperature. FBI detection can be used to clarify important parameters of the biological effects of FIR in biomedical studies and health industry applications.

## Introduction

Far-infrared radiation (FIR) has a wavelength of 3–1000 μm as suggested by the International Commission on Illumination. FIR exerts beneficial biological effects, including improving endothelial function, reducing the frequency of cardiovascular diseases, promoting wound healing, suppressing skin photoaging, and maintaining the unassisted patency of arteriovenous fistulae *in vitro* and *in vivo* studies^1–8^. Because thermal transmission always accompanies FIR emission, the biological activities of FIR are traditionally considered to be related to thermal effects^1,9^. However, a recent study revealed that the biological effect of FIR on endothelial cells is independent of thermal effects, and its effect is potentially inhibited by heat^10^. A clinical study also found that a non-thermal and long-term effect of FIR therapy plays an important role in the survival of arteriovenous fistulae in patients undergoing hemodialysis^5^. The non-thermal biological effects of FIR have potential applications in scientific research and healthcare. To date, no proper method for measuring the parameters of the biological effects of FIR, including the effective wavelengths, irradiation time, and intensity, has been developed. The development of such measurement methods will be beneficial to the clinical application of FIR.

A recent study revealed that FIR improves mitochondrial function in neuroblastoma cells^11^. Mitochondria are critical for a number of cellular functions including energy generation, calcium signaling, cell growth, and cell death^12^. Therefore, improving mitochondrial function should be one of the major mechanisms of the biological effects of FIR. Detecting mitochondria-associated cellular function should be a feasible method for detecting the biological effects of FIR. Recently, mitochondrial impairment was demonstrated to decrease bronchial epithelial cell migration^13^. Our previous study uncovered that FIR promotes HaCaT epithelial cell migration^6^. On the basis of these findings, FIR most likely promotes epithelial cell migration through upregulating mitochondrial function. In the present study, we used epithelial cell migration to establish a detection platform for quantitatively measuring the biological effects of FIR.

## Results

### The promotive effect of FIR on NRK-52E cell migration

**Figure 1:**
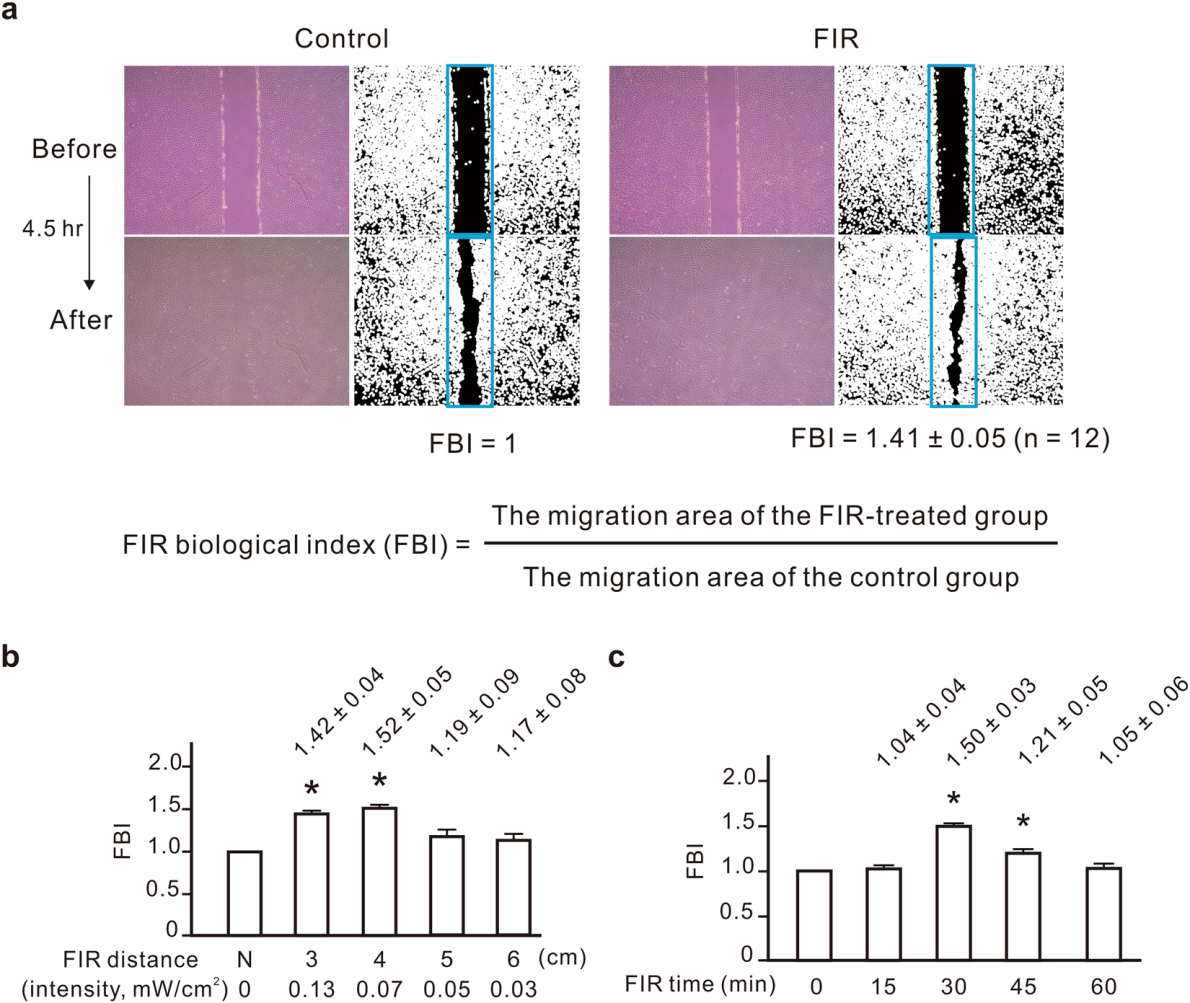
The promotive effect of far-infrared radiation (FIR) on NRK-52E cell migration. (**a**) Representative images of cell migration. NRK-52E cells were exposed to FIR (1–25 μm) for 30 min or left untreated. The distance between cells and the FIR source was 3 cm. Before calculating the migration area, the photo was first converted to a monochrome image using ImageJ. The FIR biological index (FBI) was calculated as the ratio of the FIR-promoted migration area to the migration area of the control group. (**b**) The influence of the irradiation distance of FIR on the FBI. NRK-52E cells were exposed to FIR (1–25 μm) for 30 min at the indicated distances. The FIR intensity corresponding to each distance is indicated on the horizontal axis. N, no FIR exposure. (**c**) The effective far-infrared radiation (FIR) exposure time for FIR biological index (FBI) detection. NRK-52E cells were exposed to FIR (1–25 μm) for the indicated periods at a distance of 4 cm. Results are presented as the mean ± SD (n = 6). *P < 0.05 vs. the group without FIR exposure.

To establish a detection platform for measuring the biological effects of FIR, we measured the migration of rat renal tubular epithelial cells (NRK-52E) using a ceramic full-wavelength FIR generator (1–25 μm). We found that FIR significantly promoted cell migration using our standardized method (Fig. 1a). We defined the ratio of the FIR-promoted migration area to the migration area of the control group as the FIR biological index (FBI). A higher FBI indicates a greater biological effect of FIR. Increasing the distance between the FIR irradiation source and cells is expected to reduce the intensity of FIR. After measurement and calculation, the intensity of the FIR emitter was 0.13, 0.07, 0.05, and 0.03 mW/cm^2^ at an irradiation distance of 3, 4, 5, and 6 cm, respectively. The FBI peaked at an irradiation distance of 3–4 cm and decreased at longer distances (Fig. 1b), which suggested that the most effective intensity of FIR for cell assay was 0.07 to 0.13 mW/cm^2^. These results illustrated the potential of our cell migration-based detection method for detecting the biological effects of FIR.

The effective irradiation time is also an important factor for the biological application of FIR. We detected the FBIs of different irradiation times, including 15, 30, 45, and 60 min, finding that the FBI for 30 min of irradiation was significantly higher than those of the other irradiation times (Fig. 1c). For irradiation time exceeding 30 min, the FBI declined as the irradiation time increased. Thus, the optimum irradiation time for the biological effects of FIR biological effect appears to be 30 min.

### The association of mitochondrial function with the FBI detection

**Figure 2:**
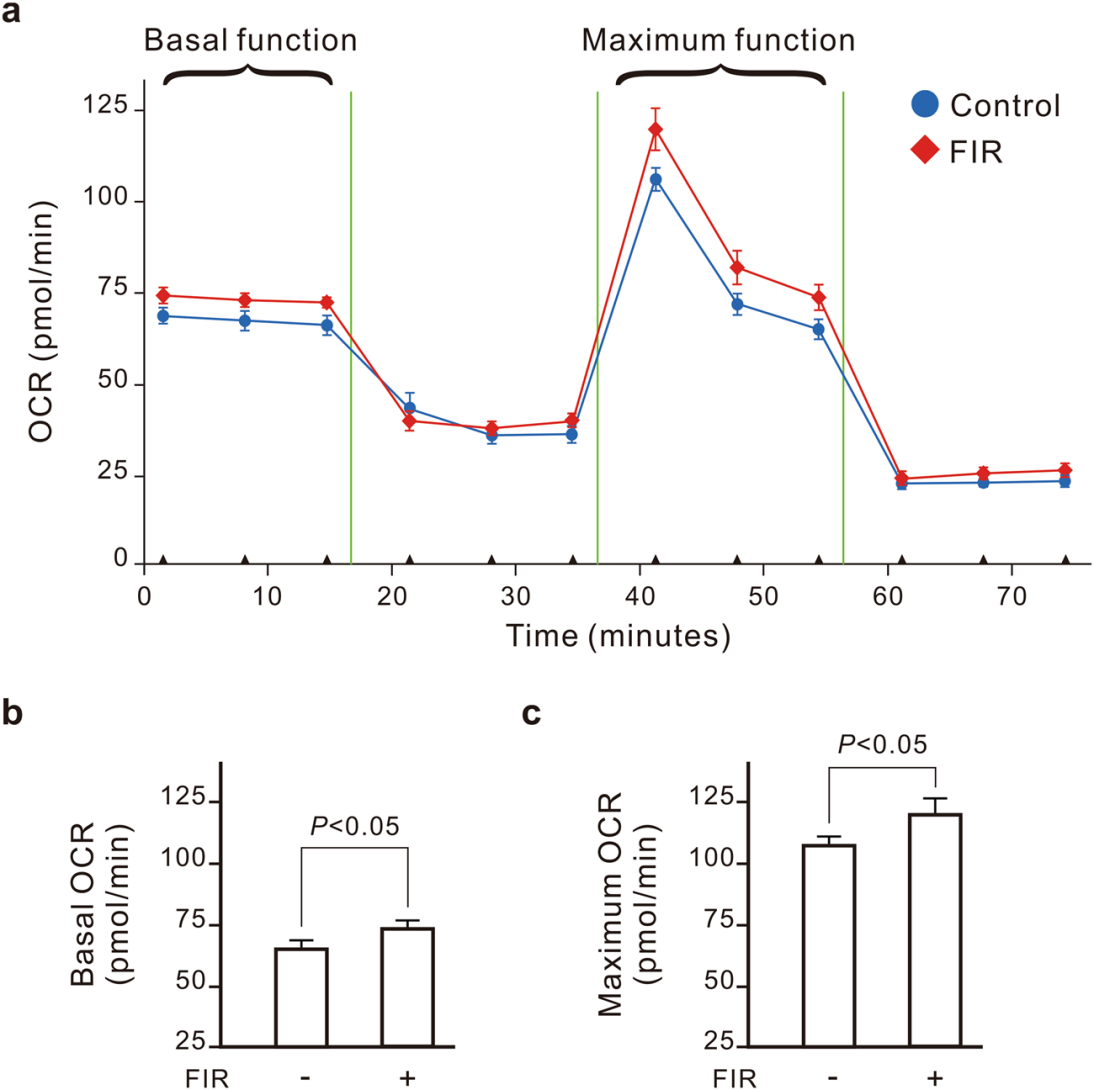
The promotive effect of far-infrared radiation (FIR) on mitochondrial function. (**a**) Oxygen consumption rate (OCR) curves. NRK-52E cells were exposed to FIR (1–25 μm) for 30 min at a distance of 4 cm or left untreated. The OCRs of the cells for the basal and maximum function areas represent the basal and maximum mitochondrial function, respectively, which are also presented as bar charts. (**b**,**c**) Results are presented as the mean ± SD (n = 3).

**Figure 3:**
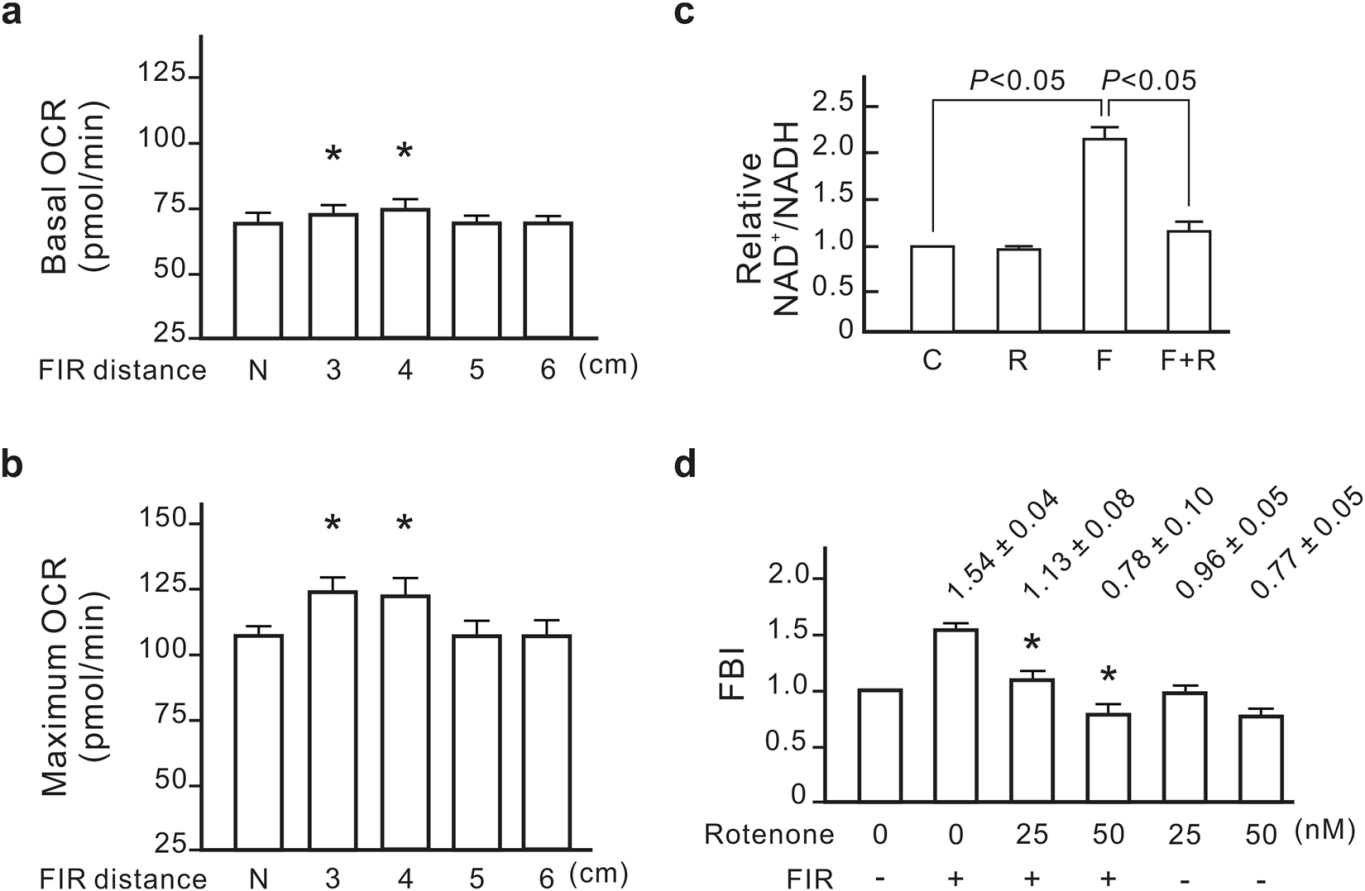
The association of mitochondrial function with far-induced radiation (FIR) biological index (FBI) detection. (**a**) The influence of FIR distances on basal oxygen consumption rates (OCRs). NRK-52E cells were irradiated with FIR (1–25 μm) for 30 min at the indicated distances. Results are presented as the mean ± SD (n = 6). N, no FIR exposure. *P < 0.05 vs. the group without FIR exposure. (**b**) The influence of FIR distances on the maximum OCR. (**c**) The promotive effect of FIR on the ratio of NDA^+^/NADH. The cells were pretreated with the mitochondria complex I inhibitor rotenone (25 nM) for 30 min and then irradiated with FIR for 30 min at a distance of 4 cm. Results are presented as the mean ± SD (n = 6). C, control; R, rotenone treatment; F, FIR irradiation. (**d**) The inhibitory effects of a mitochondria complex I inhibitor on the FBI. Results are presented as the mean ± SD (n = 6). *P < 0.05 vs. the FIR-treated group without rotenone.

We further monitored the connection between mitochondrial function and the FBI. As shown in Fig. 2, the basal and maximal levels of mitochondrial function were significantly elevated in NRK-52E cells after 60 min of FIR exposure. The lower intensity of FIR resulted in a smaller elevation of mitochondrial function (Fig. 3a,b). We also found that FIR irradiation increased the ratio of NAD^+^/NADH in NRK-52E cells (Fig. 3c). The conversion of NADH to NAD^+^ is primarily catalyzed by mitochondrial complex I. Rotenone, a mitochondrial complex I inhibitor, inhibited FIR-promoted increases in NAD^+^/NADH ratio and reduced the FBI (Fig. 3c,d). Therefore, the biological effects of FIR on mitochondria resulted in cell migration.

### The optimal cell passage number for the FBI detection

**Figure 4:**
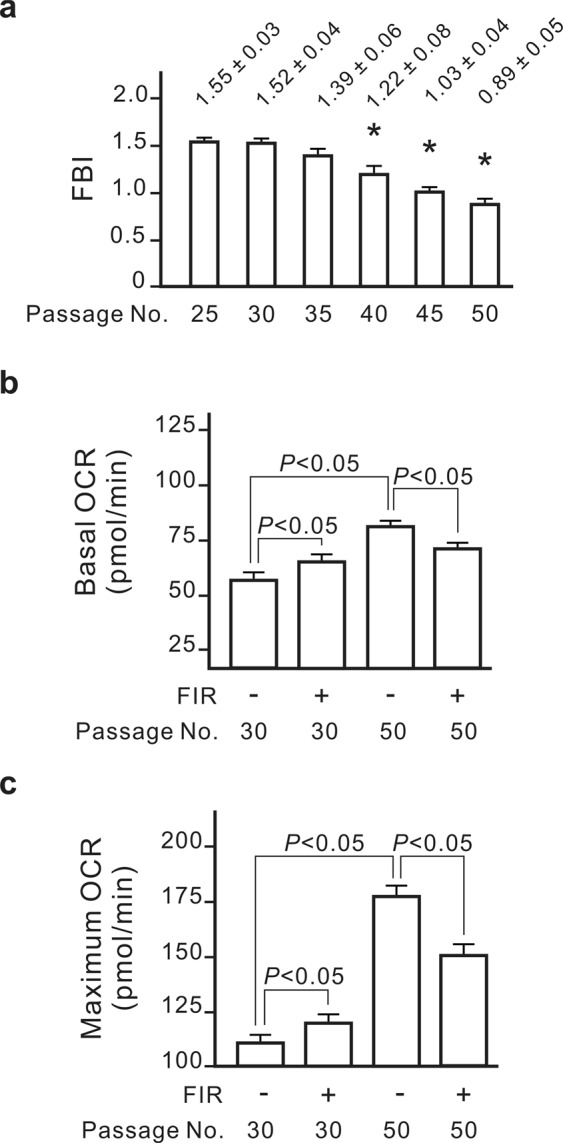
The optimal cell passage number for far-induced radiation (FIR) biological index (FBI) detection. (**a**) The influence of cell passage numbers on the FBI. NRK-52E cells with the indicated passage numbers were irradiated with FIR (1–25 μm) for 30 min at a distance of 4 cm. Results are presented as the mean ± SD (n = 6). *P < 0.05 vs. the cells at passage 20. (**b**) Basal oxygen consumption rates (OCRs) of the cells at passages 30 and 50. (**c**) Maximum OCRs of the cells at passages 30 and 50. Results are presented as the mean ± SD (n = 6).

The cell passage number may affect a cell line’s characteristics over time^14–16^. To clarify the influence of passage number on the cell migration-based detection method, we monitored the FBI in NRK-52E cells at different passage numbers. According to the cell provider (Food Industry Research and Development Institute, Taiwan), the NRK-52E cells we obtained were at passages 21. Under the same FIR conditions, NRK-52E cells expressed the highest FBI at passages 25 (P25) and P30 (Fig. 4a). The FBI gradually declined from P35 to P50. FBI was less than 1 at P50, indicating that FIR inhibits the migration of NRK-52E cells at this point. Compared with the findings at P30, both basal and maximum mitochondrial functioning were greatly increased at P50, and these functions were suppressed by FIR (Fig. 4b,c). Therefore, an NRK-52E cell passage number of less than 35 is suitable for use in our cell migration-based detection method.

### The effective FIR wavelengths for the FBI detection

**Figure 5:**
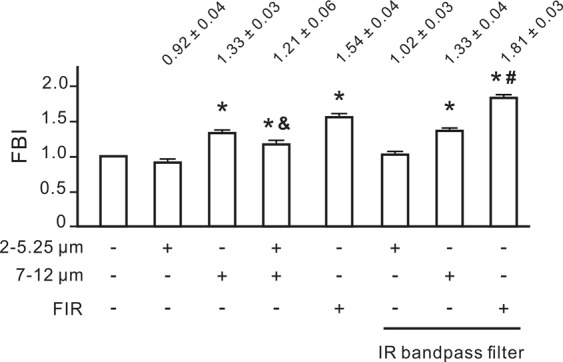
The most effective far-infrared radiation (FIR) wavelengths for FIR biological index (FBI) detection. NRK-52E cells were irradiated one full-wavelength (1–25 μm) or one of two narrowband FIR sources (2–5.25 and 7–12 μm) for 30 min. The irradiation distance was 4 cm for the full-wavelength source and 2 cm for the narrowband sources. In the groups with an IR bandpass filter, the dishes were covered with an 8- to 10-μm IR filter. Results are presented as the mean ± SD (n = 6). *P < 0.05 vs. the group without FIR exposure. ^&^*P* < 0.05 vs. the group exposed to the 7- to 12-μm FIR source. ^#^*P* < 0.05 vs. the group exposed to the full-wavelength FIR source.

**Figure 6:**
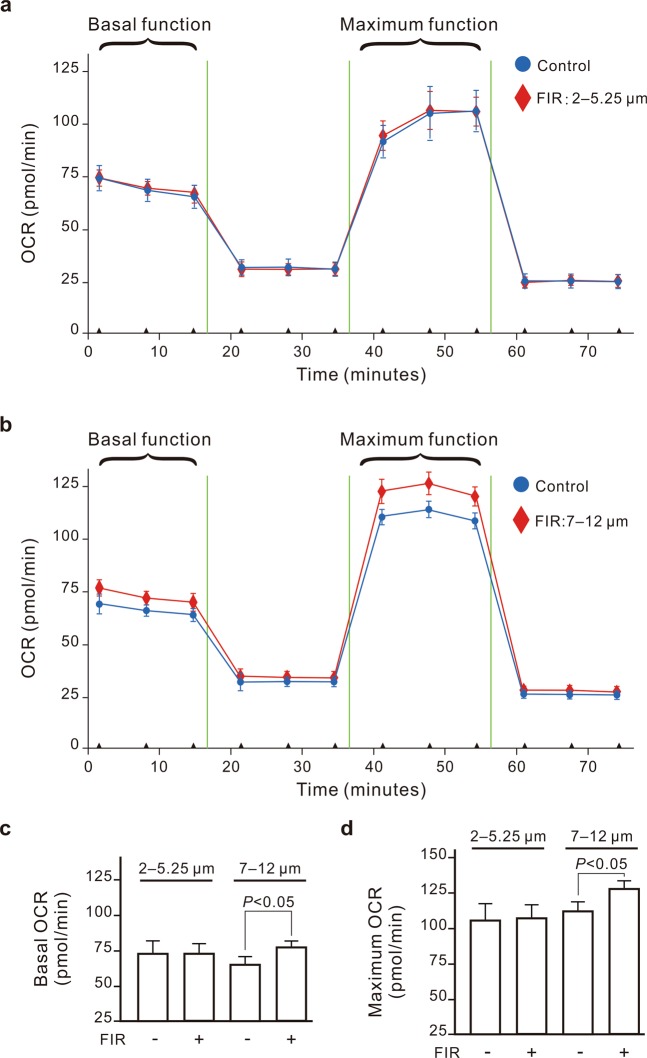
The promotive effect of narrowband FIR sources (2–5.25 and 7–12 μm) on mitochondrial function. (**a**) Oxygen consumption rate (OCR) curves of the 2- to 5.25-μm FIR source. (**b**) OCR curves of the 7- to 12-μm FIR source. NRK-52E cells were exposed to narrowband FIR sources for 30 min at a distance of 2 cm (0.07 mW/cm^2^) or left untreated. The OCRs of the cells for the basal and maximum function areas represent the basal and maximum mitochondrial function, respectively, which are also presented as bar charts. (**c**,**d**) Results are presented as the mean ± SD (n = 3).

To detect the biologically effective wavelength of FIR, we measured the biological effects of two narrowband FIR sources (2–5.25 and 7–12 μm) using our cell migration-based detection method. The intensity of two narrowband FIR sources was set at 0.07 mW/cm^2^. The 7- to 12-μm FIR source, but not the 2- to 5.25-μm source, significantly increased the FBI (Fig. 5). In fact, the 2- to 5.25-μm FIR source inhibited cell migration. We also monitored the influence of two narrowband FIR sources on mitochondrial function and found that 7- to 12-μm FIR elevated the basal and maximal levels of mitochondrial function in NRK-52E cells after 60 min of FIR exposure (Fig. 6). But 2- to 5.25-μm FIR didn’t influence the mitochondrial function (Fig. 6). To confirm the effective wavelength of FIR, an 8- to 10-μm IR bandpass filter was applied. We observed that the full-wavelength FIR source combined with the IR bandpass filter increased the FBI more strongly than did the FIR source alone (Fig. 5). Additionally, the combination of the 7- to 12-μm FIR source and the IR bandpass filter also increased the FBI. Therefore, the biologically effective wavelength of FIR is 8–10 μm.

### The inhibitory effect of heat on the biological effect of FIR

**Figure 7:**
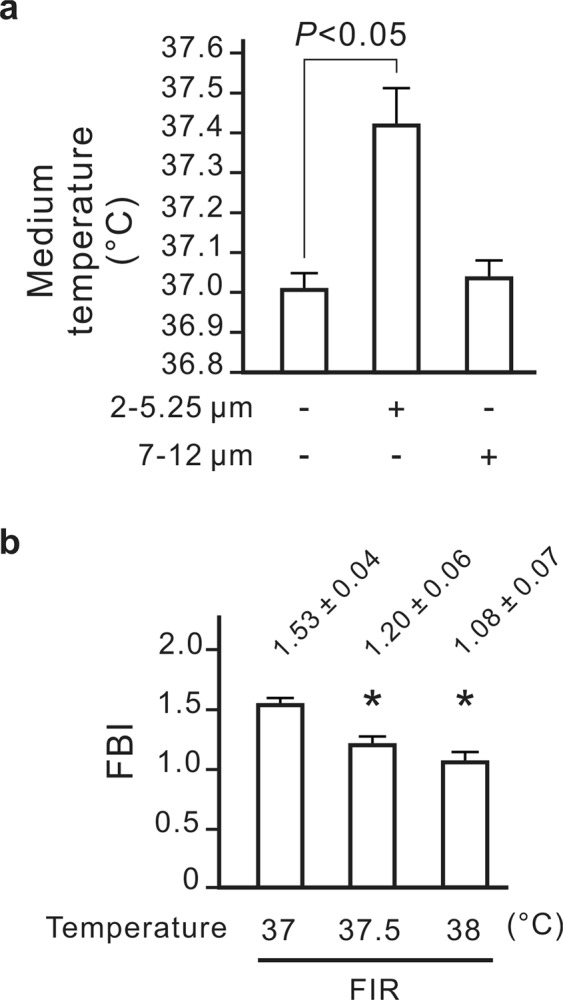
The inhibitory effect of heat on the biological effect of far-infrared radiation (FIR). (**a**) A warming effect of FIR on the culture medium. A 24-well dish containing 1 mL of culture medium per well was irradiated using one of two narrowband FIR sources (2–5.25 and 7–12 μm) for 30 min at a distance of 2 cm. Results are presented as the mean ± SD (n = 6). The 2- to 5.25-μm FIR source significantly increased the medium temperature. (**b**) The inhibitory effect of heat on the FIR biological index (FBI). NRK-52E cells were irradiated using the full-wavelength FIR source at the indicated temperatures for 30 min at a distance of 4 cm. Results are presented as the mean ± SD (n = 6). *P < 0.05 vs. the group irradiated at 37 °C.

The FBI obtained from the combination of the 2- to 5.25- and 7- to 12-μm FIR sources was lower than that obtained from the 7- to 12-μm FIR source alone (Fig. 5). In theory, a shorter FIR wavelength carries more energy and delivers more heat. After 30 min of irradiation, the 2- to 5.25-μm FIR source slightly increased the temperature of the culture medium during FBI detection, whereas the 7- to 12-μm FIR source had no such effect (Fig. 7a). To assess the influences of heat on the biological effects of FIR, the cells were cultured at difference temperatures during FIR exposure. We found that higher culture temperatures were linked to smaller FIR-induced increases of the FBI (Fig. 7b). Therefore, our data revealed that heat inhibits the biological effects of FIR.

## Discussion

In recent years, many studies have examined the effects of FIR on human health and cell physiology^1–8^, which suggest that FIR have great potential for application in health care. However, the biological effects of FIR that have been discovered so far are difficult to be quantitatively analyzed. There has been a lack of a quantitative analyze method for the biological effects of FIR, which leads to uncertainty in the application parameters of FIR. Since the promotive effect of FIR on mitochondrial function was discovered^11^, it become possible to quantitatively measure the biological effects of FIR. But the measurement of mitochondrial function is costly and complicated. Epithelial cell migration is highly associated with mitochondrial function^17,18^. Mitochondrial fission has been found to be necessary for proper lymphocyte migration^19^. We found that the expression of dynamin-related protein 1 (Drp1), a mitochondrial fission-associated protein, was upregulated in NRK-52E cells 60 min after FIR exposure (Supplementary Fig. S1). The expression of Mfn2, a mitochondrial fusion-associated protein, was also decreased slightly 2 h after FIR exposure. That finding supports the connection between FIR irradiation and mitochondria-mediated cell migration in NRK-52E cells. Therefore, we established a cell migration-based detection method for quantitively measuring the biological effects of FIR. Using the FIR biological index (FBI), we confirmed that an increase of the FBI was linked to FIR-promoted increase of mitochondrial function. The FBI detection method is more efficient and less expensive than a detection method based on mitochondrial function. As shown in this study, the FBI detection method is potent to measure the biological effects of FIR under different environmental conditions. Therefore, the FBI detection method is potential to be applied to the condition setting of FIR research and the development platform of the FIR commodities.

The biological effects of FIR that have been discovered on mammalian cells are variable^1–8^. Whether the FBI detection method is applicable in all systems is still uncertain. Our results show that FBI detection method is based on mitochondrial function upregulation. FIR irradiation upregulated the activity of mitochondrial complex I and then increased the ratio of NAD^+^/NADH (Fig. 3). The ratio of NAD^+^/NADH affects many important physiological phenomena in mammalian cells. For example, the increase of the NAD^+^/NADH ratio activates Sirtuin 1 (Sirt1), which targets numerous regulatory factors affecting stress management, metabolism and aging^20^. The NAD^+^/NADH ratio also regulates the binding activity of the corepressor CtBP (carboxyl-terminal binding protein), which is involved in transcriptional pathways important for development, cell cycle regulation, and transformation^21^. In other words, the biological effect of FIR identified by the FBI detection method is potential to influence NAD^+^/NADH-regulated physiological phenomena. Therefore, we suggest that the FBI detection method is applicable in the biological effects of FIR associated with mitochondrial function and the NAD^+^/NADH ratio change.

FBI detection is a simple and stable method for measuring the biological effects of FIR. Our data showed that the difference between repeated experimental groups is relatively small (S.D. <0.1) (Figs 1, 3, 4, 5 and 7). The most important factor affecting the stability of the FBI detection method is the passage number of the cells. High passage numbers often represent senescence of the cells and affect a cell line’s phenotypes and characteristics^22,23^. NRK-52E is a non-transformed cell line and sensitive to senescence. Our results suggested that the mitochondrial function abnormally increased as the passage number of NRK-52E increased. Because mitochondrial function was linked to the FBI detection, FBI was gradually decreasing as the passage number increased (Fig. 4). Therefore, the optimal passage number of NRK-52E cells is less than 35 for use in the FBI detection as shown in Fig. 4a.

The biologically effective wavelength of FIR has long been a mystery although there are many studies investigating the biological effects of FIR. Using narrowband FIR sources and an IR bandpass filter, we found that the effective biologically effective wavelength of FIR is between 8 and 10 μm (Fig. 5). In addition, a shorter FIR wavelength (2 to 5.25 μm) instead inhibited the biological effects of FIR. This may result from that the shorter wavelengths will transfer higher heat. Heat inhibited FIR-induced increases of the FBI as shown in Fig. 7. Our previous study also revealed that heat inhibited FIR-induced nuclear translocation of promyelocytic leukaemia zinc finger protein in endothelial cells^4^. Most commercially available FIR emitters use high temperatures to produce high-intensity FIR, which is detrimental to the biological effects of FIR. In fact, the full-wavelength FIR source increased the FBI more strongly after retaining only the 8- to 10-μm wavelength (Fig. 5). Our study provides the first evidence of the biologically effective wavelengths of FIR and the inhibitory effects of shorter wavelengths of FIR on the biological effects of FIR.

In summary, we established a method using rat renal tubular cells for measuring the biological effects of FIR. The principle of FBI detection is highly correlated with FIR-promoted mitochondrial function. However, the FBI detection method is more efficient and less expensive than a detection method based on mitochondrial function. Our measurements identified the most effective irradiation time for FIR as 30 min. The most biologically effective wavelength of FIR was 8–10 μm. Our data also reveal that short-wavelength FIR (2–5.25 μm) transmits more heat, which in turn inhibits the biological effects of FIR. FBI detection will contribute to the study and application of FIR in biomedical research.

## Methods

### Cell migration-based detection of the biological effects of FIR

NRK-52E cells were purchased from the Food Industry Research and Development Institute (Taiwan). The cells were cultured in DMEM supplemented with an antibiotic/antifungal solution and 17% fetal calf serum (FCS) in a CO_2_ incubator at 37 °C and 95% humidity. Cells were used between the 20th and 35th passage unless specified. In total, 5.6 × 10^3^ cells/well were seeded into a Culture-Insert 2 Well (ibidi GmbH, Martinsried, Germany) and cultured for 16 h. After removing the Culture-Inserts, we took micrographs (×40) of the cells, which were used as the starting pictures of a cell migration assay. The cultured cells were irradiated via FIR from various sources in a CO_2_ incubator under the indicated conditions, incubated for 4 h, and then photomicrographed to prepare the ending pictures of the cell migration assay. The wound areas in the pictures were measured using ImageJ, and conclusions of the cell migration area were based on the difference between the wound areas in the starting and ending pictures. We identified the FBI as the ratio of the migration area of the FIR-treated group to that of the control group.

### FIR sources

The PDA-3258 FIR emitter ceramic full-wavelength FIR generator is manufactured by Ever Spring Advanced (Tainan, Taiwan). The FIR average emissivity of the device is 0.935, and the output power is 5–7 W at 50 °C. The emission wavelength covers 1–25 μm. In our study, the operating temperature of the FIR generator was 40 °C. Two narrowband FIR sources, NM5NSC (2–5.25 μm) and NL5NGC (7–12 μm), were also used in this study and purchased from ICX Photonics (Billerica, MA, USA). The maximum input power of the two sources is 2 W. The two sources were driven at 3 V with a 1 Hz pulsed operation. An 8- to 10-μm IR bandpass filter (Part No. 9000/450-60177-B, Center wavelength: 9000 ± 50 nm, Bandwidth: 450 ± 50 nm, Transmission: 85%) was purchased from Andover Co. (Salem, NH, USA). The transmission spectrum of the filter is shown in Supplementary Fig. S2. The intensity of the FIR sources was calculated from a reference FIR source (TY301 FIR emitter, WS Far Infrared Medical Technology, Taipei, Taiwan), which was detected and calculated by China National Infrared & Industrial Electrothermal Products Quality Supervision & Test Center (Wuhan, China)^10^. The PDA-3258 FIR emitter has an intensity of 0.13 mW/cm^2^ at an irradiation distance of 3 cm and the intensity of NM5NSC (2–5.25 μm) and NL5NGC (7–12 μm) was 0.07 mW/cm^2^ at an irradiation of 2 cm. In the temperature tests, the temperature of culture medium was detected using an infrared thermometer GM320 (Temperature range: −50 ~380 °C, Accuracy: ±1.5%, Resolution: 0.1 °C, Jumaoyuan Science and Technology Co., Shenzhen, China).

### Mitochondrial function assay

NRK-52E cells (3 × 104 cells/well) were seeded in a Seahorse XFp Cell Culture Miniplate (Agilent Technologies, Santa Clara, CA, USA) in normal condition medium for 16–18 h. After FIR exposure, the cell medium was replaced with conditional medium (culture medium with 2% FCS but lacking sodium bicarbonate) and incubated without supplied CO_2_ for 1 h before the completion of probe cartridge calibration. To monitor mitochondrial function, we detected the oxygen consumption rate using a Seahorse XFp analyzer (Agilent Technologies) according to the manufacturer’s recommended protocol. Upon completion of the Seahorse XFp analysis, cells were lysed to calculate the protein concentration using the BCA assay (Pierce Biotechnology, Rockford, IL, USA). The results were normalized to the protein optical density values of the corresponding wells. Rotenone was obtained from Sigma-Aldrich (St. Louis, MO, USA).

### NAD^+^/NADH ratio detection

NAD^+^/NADH ratio was measured using a NAD/NADH Quantitation Colorimetric kit purchased from BioVision (Milpitas, CA) according to the manufacturer’s instructions.

### Statistical analyses

Data were presented as the mean ± SD. The differences between two groups were determined using Student’s t-test, and *P* < 0.05 was considered statistically significant.

## Supplementary information

Supplementary Information
