# Supplementary material for: Detecting the limits of the biological effects of far-infrared radiation on epithelial cells
Source: Sci Rep. 2019 Aug 12;9:11586. doi: 10.1038/s41598-019-48187-0 (PMC6690987; doi:10.1038/s41598-019-48187-0)
Supplement: Supplementary file 1 — Supplementary Information [file 41598_2019_48187_MOESM1_ESM.pdf]

Detecting the limits of the biological effects of far-infrared radiation on epithelial cells

Yung-Ho Hsu[1,2], Yu-Wei Chen[2], Chung-Yi Cheng[1,3], San-Liang Lee[4], Tzu-Hsuan Chiu[2], Cheng-Hsien Chen[1,2,3]*

[1] Department of Internal Medicine, School of Medicine, College of Medicine, Taipei Medical University, Taiwan.
[2] Division of Nephrology, Department of Internal Medicine, Shuang Ho Hospital, Taipei Medical University, Taiwan.
[3] Division of Nephrology, Department of Internal Medicine, Wan Fang Hospital, Taipei Medical University, Taiwan.
[4] Department of Electronic and Computer Engineering, National Taiwan University of Science and Technology, Taiwan.

*Correspondence to hippy@tmu.edu.tw

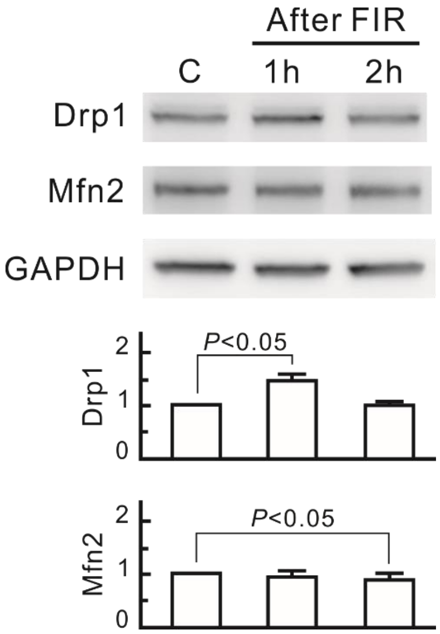

Supplementary Figure S1. The Western blots of Drp1 and Mfn2. NRK-52E cells were irradiated with FIR (1–25 µm) for 30 min at 0.07 mW/cm^2. The cells were harvested 1 or 2 h after FIR irradiation and a total of 15 µg of NRK-52E protein lysate was applied to each lane and analyzed by for Western blotting. GAPDH was detected as a loading control. Relative levels of the protein bands were quantified from five independent experiments by Quantiscan software (Biosoft, Cambridge, United Kingdom) and presented in bar chart forms. Results are expressed as the mean ± SD (n = 5). The original blots are shown in Supplementary Figure S3.

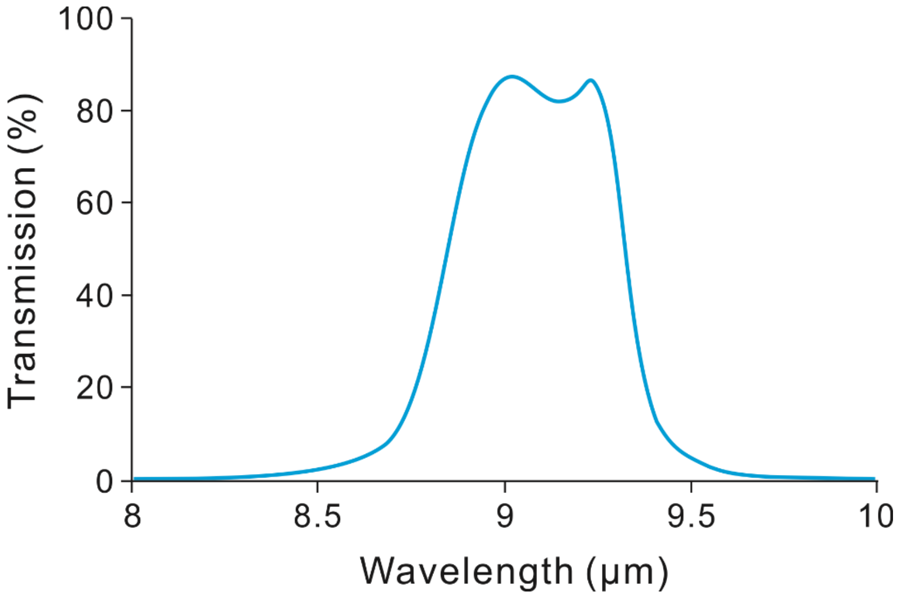

Supplementary Figure S2. SURPLUS 9000/450-60177-B transmission spectrum.

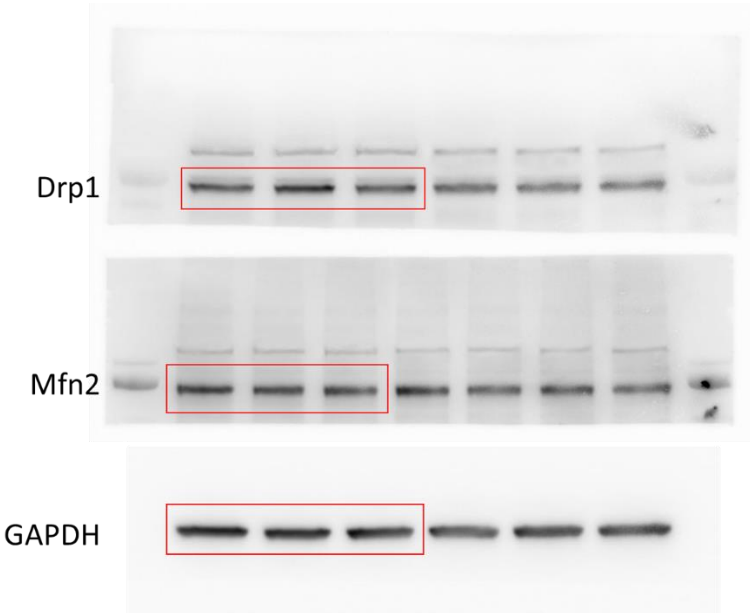

Supplementary Figure S3. The original blots in Supplementary Figure S1.
